# SOXC Enhances NGN2‐Mediated Reprogramming of Glioblastoma Cells Into Neuron‐Like Cells by Modulating RhoA and RAC1/CDC42 Pathway Activity

**DOI:** 10.1111/cns.70075

**Published:** 2024-10-10

**Authors:** Jianjing Yang, Xiaohong Zhu, Fan Wang, Zhen Chen, Ying Zhang, Jiawei Chen, Haoqi Ni, Chun‐Li Zhang, Qichuan Zhuge

**Affiliations:** ^1^ Department of Neurosurgery The First Affiliated Hospital of Wenzhou Medical University Wenzhou Zhejiang China; ^2^ Zhejiang‐US Joint Laboratory for Aging and Neurological Disease Research The First Affiliated Hospital of Wenzhou Medical University Wenzhou China; ^3^ Zhejiang Provincial Key Laboratory of Aging and Neurological Disorder Research The First Affiliated Hospital of Wenzhou Medical University Wenzhou China; ^4^ Department of Molecular Biology University of Texas Southwestern Medical Center Dallas Texas USA; ^5^ Hamon Center for Regenerative Science and Medicine University of Texas Southwestern Medical Center Dallas Texas USA

**Keywords:** ECT2, glioblastoma, NGN2, RAC1/CDC42, RhoA, SOX4/11

## Abstract

**Background:**

Glioblastoma represents the most frequently diagnosed malignant neoplasm within the central nervous system. Human glioblastoma cells can be phenotypically reprogrammed into neuron‐like cells through the forced expression of NEUROG2 and SOXC factors. NEUROG2 serves as a pioneer factor, establishing an initial framework for this transformation. However, the specific role of SOXC factors has not been fully elucidated.

**Methods:**

In this study, we used ChIP‐seq to determine the potential target gene of NGN2. RNA‐seq has been used to evaluate the transcriptional change during NGN2‐SOX11‐mediated neuron reprogramming. Immunofluorescence was used to determine the neuron reprogramming efficacy and cell proliferation ability. ChIP‐qPCR, Co‐IP, and Western Blot were performed to investigate the mechanism.

**Results:**

Our findings reveal that SOXC factors, in contrast to their previously identified function as transcriptional activators, act as transcriptional repressors. They achieve this by recruiting TRIM28 to suppress the expression of ECT2, a RhoGEF. This suppression results in the differential regulation of RhoA, RAC1, and CDC42 activities throughout the reprogramming process. We further establish that small molecules targeting RhoA and its effectors can substitute for SOXC factors in facilitating the neuronal reprogramming of glioblastoma cells.

**Conclusion:**

These results underscore the pivotal role of SOXC factors' transcriptional repression and illuminate one of their specific downstream targets.

## Graphical abstract

SOXC factors identified as transcriptional activators, act as transcriptional repressors, in glioblastoma. They achieve this by recruiting TRIM28 to suppress the expression of ECT2, a RhoGEF. This suppression results in the differential regulation of RhoA, RAC1, and CDC42 activities throughout the reprogramming process.

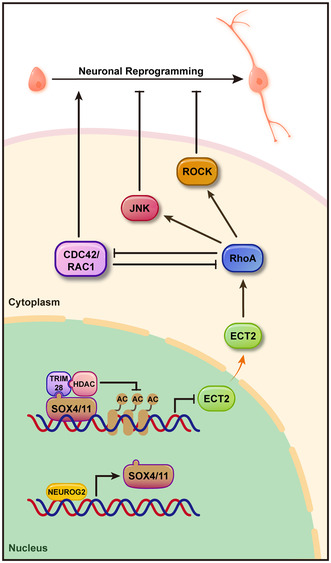

## 1 Introduction

Glioblastoma is the most common and deadly primary tumor in the central nervous system [1]. One salient feature of glioblastoma cells is their resistance to terminal differentiation. We and others have recently demonstrated that these cells can be phenotypically reprogrammed into non‐proliferative and terminally differentiated neuron‐like cells [2, 3, 4, 5, 6]. Such fate change can be accomplished by forced expression of master regulators of neurogenesis. One of these factors is NGN2 (also known as NEUROG2), which can convert astroglia and fibroblasts to neurons [7, 8, 9] and functions as a pioneer factor during the reprogramming process [10]. Although it alone can induce differentiation of glioma stem‐like cells [4], NGN2 requires SOX4 or SOX11 for highly efficient neuronal reprogramming of human glioblastoma cells [3, 10]. It is not understood, however, how SOX4 or SOX11 facilitates this fate switch.

SOX4 and SOX11 belong to the SOXC subfamily of Sry‐related high mobility group (HMG) box (SOX) superfamily of transcription factors [11, 12]. They are highly expressed during neural development and in the adult neurogenic niche [13, 14, 15, 16, 17, 18]. Although germline deletion of either SOX4 or SOX11 results in embryonic lethality due to heart defects, the central nervous system is largely unaffected in these single gene‐deletion mutant mice indicating genetic redundancy [17, 18]. Indeed, neuronal differentiation is almost completely inhibited when SOX4 and SOX11 are simultaneously knocked down in the developing chick spinal cord [14] or conditionally deleted in the adult mouse hippocampus [19]. They are also redundantly required for normal organogenesis during early development [20]. Examination of target genes indicates that SOXC factors mainly function as transcriptional activators to promote expression of neuronal genes or genes in the Hippo pathway [13, 14, 19, 20]. It remains unclear whether and how they may also serve as transcriptional repressors in the nervous system.

The Rho family of GTPases, including CDC42, RAC1, and RhoA, play fundamental roles in neural development, plasticity, regeneration, and diseases [21, 22, 23, 24, 25, 26, 27]. The Rho GTPases cycle between two states: an active GTP‐bound state and an inactive GDP‐bound state. The guanosine exchange factors (GEFs) catalyze the exchange of GDP for GTP to activate Rho proteins, which are then inactivated by GTPase‐activating proteins (GAPs) by promoting hydrolysis of GTP to GDP. One of the RhoGEFs is ECT2 (epithelial cell transforming sequence 2), which is essential for cytokinesis and cell migration [28, 29]. It is regulated not only by subcellular localization and phosphorylation [28, 30, 31, 32] but also at the transcriptional level. Overexpression of ECT2 is associated with glioblastoma and promotes proliferation and invasion of glioma cells [33, 34, 35, 36, 37, 38].

In this study, we examined the molecular mechanism by which SOX4 or SOX11 enables NGN2 to highly efficiently reprogram human glioblastoma cells into neurons. We focused on downregulated genes during the reprogramming process, since they represent a large fraction of differentially expressed genes mediated by SOX11. Furthermore, it was less clear whether SOXC factors could function as transcription repressors controlling gene expression. Our results revealed that ECT2 is a key target that is directly suppressed by SOX4/11 through the recruitment of TRIM28 corepressors. Consistent with ECT2 as a RhoGEF, the activity of RhoA, RAC1, and CDC42 is also differentially altered during the fate reprogramming process. Most importantly, the function of SOXC factors can be replaced by chemicals targeting RhoA, RAC1, CDC42, or their effectors, indicating a critical role of Rho GTPases played during neuronal reprogramming of human glioblastoma cells.

## 2 Results

### 2.1 SOXC Factors are Essential for NGN2‐Mediated Fate Reprogramming of Human Glioblastoma Cells

**FIGURE 1:**
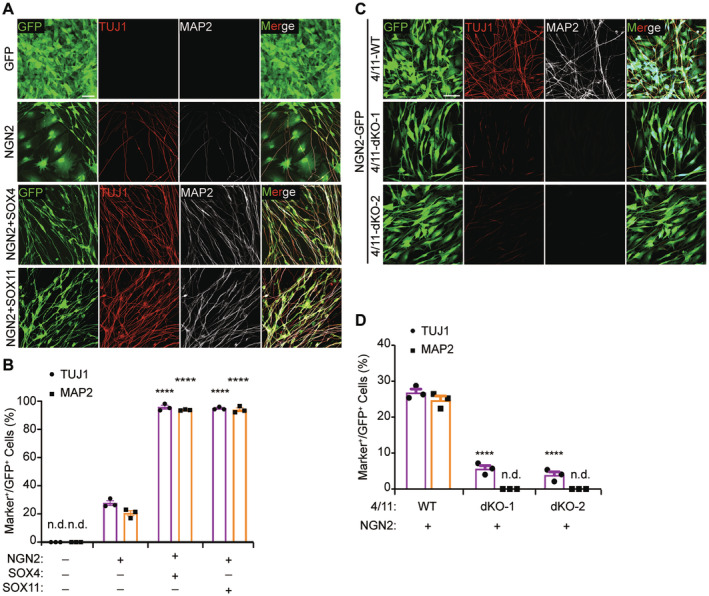
SOXC factors are essential for NGN2‐mediated fate reprogramming of human glioblastoma cells. (A) The efficiency of reprogramming has been assessed using immunocytochemistry, employing the neural markers TUJ1 and MAP2. This evaluation was conducted 12 days postinfection with GFP, GFP‐NGN2, GFP‐NGN2‐SOX4, and NGN2‐SOX11 lentivirus in the U251 cell line. (B) Quantification of Tuj1 and Map2 positive cells, which were normalized to GFP positive cell (mean ± SEM; *n* = 3; ****p < 0.0001; n.d., non detectable). (C) Quantification of Tuj1 and Map2 positive cells, which was normalized to GFP positive cell (mean ± SEM; *n* = 3; ****p < 0.0001; dKO, double knockout cell lines; n.d., non detectable). (D) The efficiency of reprogramming, mediated by NGN2, has been evaluated in the SOX4/11 double knockout cell lines. This assessment was conducted using the neural markers TUJ1 and MAP2, following the infection of the GFP‐NGN2 lentivirus at 12 days postinfection (dpi) in U251 cells (Scale bar = 50 μm).

We first confirmed our previous findings that NGN2, as a pioneer factor, could reprogram U251 human glioblastoma cells into neurons that are marked by expression of TUJ1 and MAP2 [3, 10]. When examined at 12 days post‐virus infection (dpi), approximately 20% of NGN2‐expressing U251 cells dramatically changed into bipolar or multipolar elongated neuronal morphology and robustly expressed TUJ1 and MAP2, whereas the control GFP‐expressing cells divided rapidly and became confluent during this period (Figure 1A). The inclusion of either SOX4 or SOX11 remarkably promoted morphological transformation and increased the reprogramming efficiency to about 95% measured by the ratio of cells expressing TUJ1 and MAP2 (Figure 1A,B). By 21 dpi, nearly 35% of the converted MAP2‐positive cells expressed NeuN, a marker for mature neurons (Figure S1A–C). In addition to U251 glioblastoma cells, we also examined primary glioblastoma stem cells (GBM) directly isolated from human glioblastoma brains. The results showed that NGN2 and SOX4/11 could reprogram primary glioblastoma cell lines (GBM) into neuron‐like cells with approximately 95% efficiency, as measured by the ratio of cells expressing TUJ1 and MAP2 (Figure S2A–D).

ChIP‐seq showed that SOX4 and SOX11 are the downstream target genes of NGN2 (Figure S3A). Besides, RNA‐seq and Western blotting showed that both SOX4 and SOX11 were basally expressed in U251 cells and were further induced by NGN2 (Figure S3B,C). Such induction of SOXC factors might contribute to the observed low but significant reprogramming of U251 cells by NGN2 alone. To test this possibility, we made U251 cells lacking both SOX4 and SOX11 through CRISPR/Cas9‐mediated gene editing. Western blotting confirmed complete deletion of SOX4 and SOX11 in double knockout (dKO) cells (Figure S3E). When these cells were transduced with NGN2‐expressing lentivirus and examined at 12 dpi, MAP2‐expression was not detectable (Figure 1C,D). The number of TUJ1+ cells was also greatly reduced from 28% in SOX4/11‐wild type cells to about 5% in dKO cells (Figure 1C,D). These residual TUJ1 + dKO cells expressed much lower level of TUJ1 and failed to exhibit neuron‐like morphology (Figure S3D). Together, these results indicate that SOX4 and SOX11 are essential for NGN2‐mediated fate reprogramming of U251 cells. Of note, SOX4 or SOX11 alone was not sufficient to induce fate reprogramming, consistent with our previous results showing NGN2 serves as a pioneer factor during the reprogramming process [10].

### 2.2 SOXC Factors Do Not Globally Influence NGN2 Activity

**FIGURE 2:**
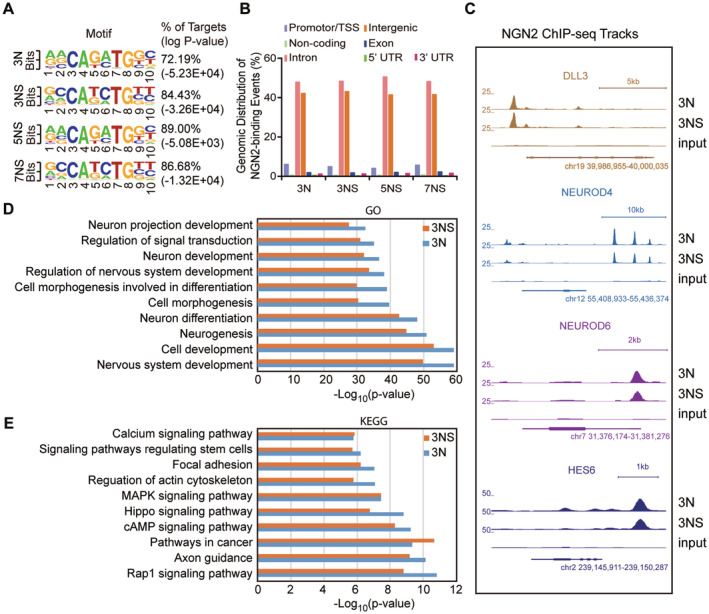
NGN2 ChIP‐seq analysis has been conducted to identify genes targeted by NGN2 in the U251 cell line. (A) The NGN2‐binding motif has been identified through NGN2 ChIP‐seq analysis across various groups (3 N, 3 days post NGN2 lentivirus infection; 3NS, 3 days post NGN2‐SOX11 lentivirus infection; 5NS, 5 days post NGN2‐SOX11 lentivirus infection; 7NS, 7 days post NGN2‐SOX11 lentivirus infection). (B) The genome‐wide distribution of NGN2 binding sites has been analyzed across various groups. (C) The binding track of NGN2 for the DLL3, NEUROD4, NEUROD6, and HES6 genes has been analyzed across various groups. (D) The gene‐ontology analysis has been conducted for the biological processes of genes targeted by NGN2. (E) The analysis of key signaling pathways has been conducted for genes targeted by NGN2.

To understand how SOXC factors enable NGN2 to efficiently reprogram U251 cells, we first examined whether NGN2 function was globally influenced by SOX11. This was determined by ChIP‐seq in U251 cells transduced with lentivirus expressing either NGN2 alone or in combination with SOX11 during a time course. A consensus NGN2‐binding motif is E‐box. This represented about 72% of all NGN2‐binding events in U251 cells expressing NGN2 alone (Figure 2A). The inclusion of SOX11 did not greatly alter this consensus NGN2‐binding motif, which occupied about 84% of all NGN2 targets in U251 cells expressing NGN2 and SOX11. Genome‐wide NGN2‐binding events were detected overwhelmingly in intergenic and intronic regions, and such distribution patterns remained unchanged by inclusion of SOX11 (Figure 2B). We also examined in detail a few NGN2 targets, such as DLL3, NEUROD4, NEUROD6, and HES6. NGN2‐binding on these targets was clearly not influenced by SOX11 (Figure 2C). Gene‐ontology analysis of biological processes showed that NGN2 targets were mainly involved in nervous system development and neurogenesis (Figure 2D), consistent with its well‐established role in specifying neuronal fate during development [39]. The NGN2 targets were also key components of several key signaling pathways, such as Rap1 signaling, axon guidance, Hippo pathway, and regulation of actin cytoskeleton (Figure 2E). All these NGN2‐targeted signaling pathways and biological processes were not markedly changed by ectopic SOX11 in U251 cells.

### 2.3 RNA‐Seq Implicates a Role of SOXC Factors in Gene Repression

**FIGURE 3:**
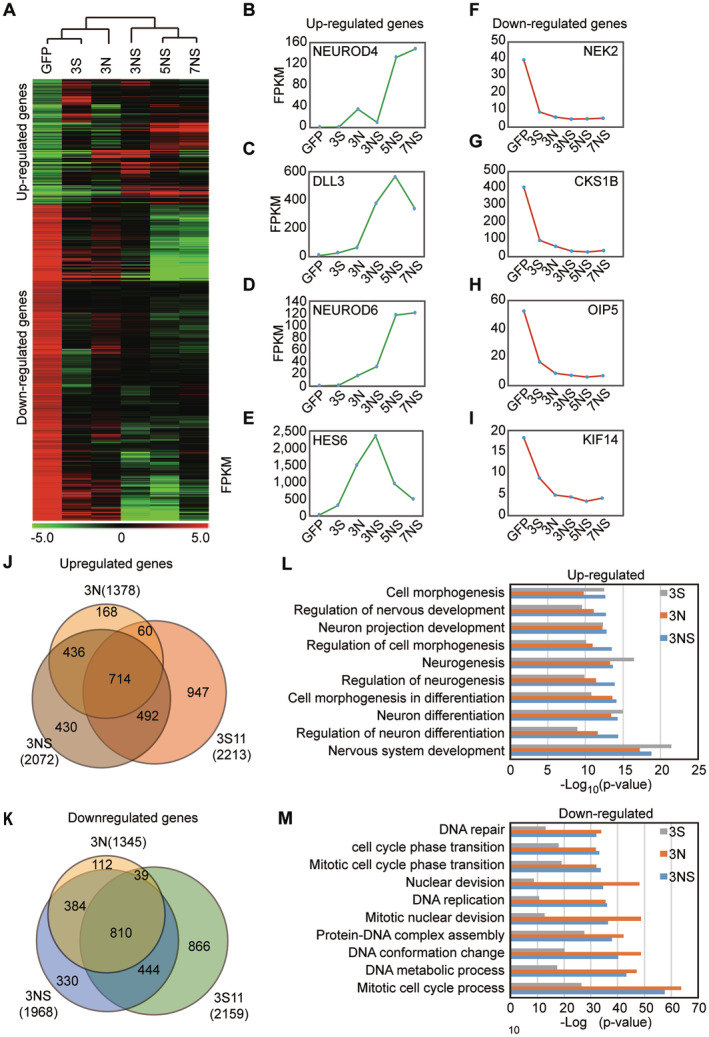
RNA‐seq is utilized to identify changes in the transcriptome during the reprogramming process. (A) Hierarchical clustering and a heatmap are used to visualize changes in transcription detected by RNA‐seq, compared to the control group. In the heatmap, green signifies a decrease in gene expression, while red indicates an increase (*n* = 3) (B–E) The mainly up‐regulated genes (NEUROD4, DLL3, NEUROD6, and HES6) in different groups. (F–I) The mainly down‐regulated genes (HEK2, CKS1B, OIP5, and KIF14) in different groups. (J, K) Venn diagram showed the overlapping genes between different group (3 N, 3NS, 3S), whose expression fold changes are more 2‐fold or less than −2‐fold identified in each group. (L) Gene ontology (GO) analysis is conducted to explore the biological functions of the up‐regulated genes. (M) Gene ontology (GO) analysis is conducted to explore the biological functions of the down‐regulated genes.

We next explored the influence of SOXC factors on global gene expression by RNA‐seq. U251 cells were transduced with lentivirus expressing GFP, SOX11, NGN2, or a combination of NGN2 and SOX11 during a time course. Biological triplicates were processed for RNA‐seq. Unbiased hierarchical clustering clearly showed progressive fate change from GFP‐expressing U251 cells to those expressing both NGN2 and SOX11 at 7 dpi (Figure 3A). When compared to the GFP control, a total of 280 and 705 genes were significantly up‐regulated and down‐regulated, respectively, under all culture conditions (Figure 3A; Table S1). Consistent with morphological changes, the up‐regulated genes were mainly involved in neuron differentiation and nervous system development, such as NEUROD4, DLL3, NEUROD6, and HES6 (Figure 3B–E). On the other hand, the down‐regulated genes played critical roles in protein‐DNA complex assembly, chromatin and nucleosome assembly, and mitotic cell cycles. Examples of these genes were HEK2, CKS1B, OIP5, and KIF14 (Figure 3F–I). This large number of down‐regulated genes explained cell cycle exit of rapidly dividing U251 cells during the fate reprogramming process mediated by SOX11 and NGN2. Consistently, U251 cell proliferation was directly assessed using Ki67 staining and BrdU incorporation assays. The expression of NGN2 and/or SOX11 significantly reduced the proportion of U251 cells positive for Ki67 and BrdU staining across all analyzed time points (Figure S4A–F). Additionally, the total ATP content in viable cells ceased to increase following NGN2 and/or SOX11 expression in U251 (Figure S4G). These were in sharp contrast to the control GFP expressing U251, which in general doubled viable cells from 3 to 7 dpi. Together, the above results clearly indicate that NGN2 and SOX11 potently inhibit growth and proliferation of human glioblastoma cells and may serve as a tumor suppressor for this type of malignancy.

We then focused our analysis on 3 dpi to understand the early events that could explain the critical role of SOXC factors. Venn diagrams have been used to compare the transcriptome differences among the 3 N, 3S, and 3NS groups. Our findings revealed that a substantial portion of the upregulated genes in the 3S group are also present in the 3 N group (390/761) and the 3NS group (617/1163) (Figure 3J). Similarly, among the downregulated genes, the 3S group encompasses 924 of the 1357 genes in the 3 N group and 975 of the 1752 genes in the 3NS group (Figure 3K). These Venn diagrams suggest that SOX11 may have a function analogous to that of NGN2, as both can induce similar changes in gene expression during reprogramming. Besides, we conducted a gene‐ontology (GO) analysis using DAVID to scrutinize the upregulated and downregulated genes. Our analysis revealed that the primary biological functions of these upregulated genes are mainly associated with nervous system development, regulation of neuron differentiation, neuron differentiation, and cell morphogenesis in differentiation (Figure 3L). Conversely, GO analysis of the downregulated genes indicated that their main biological functions are related to the mitotic cell cycle process, DNA metabolic process, and DNA conformation change (Figure 3M).

### 2.4 SOXC Factors Recruit TRIM28 to Directly Repress ECT2

**FIGURE 4:**
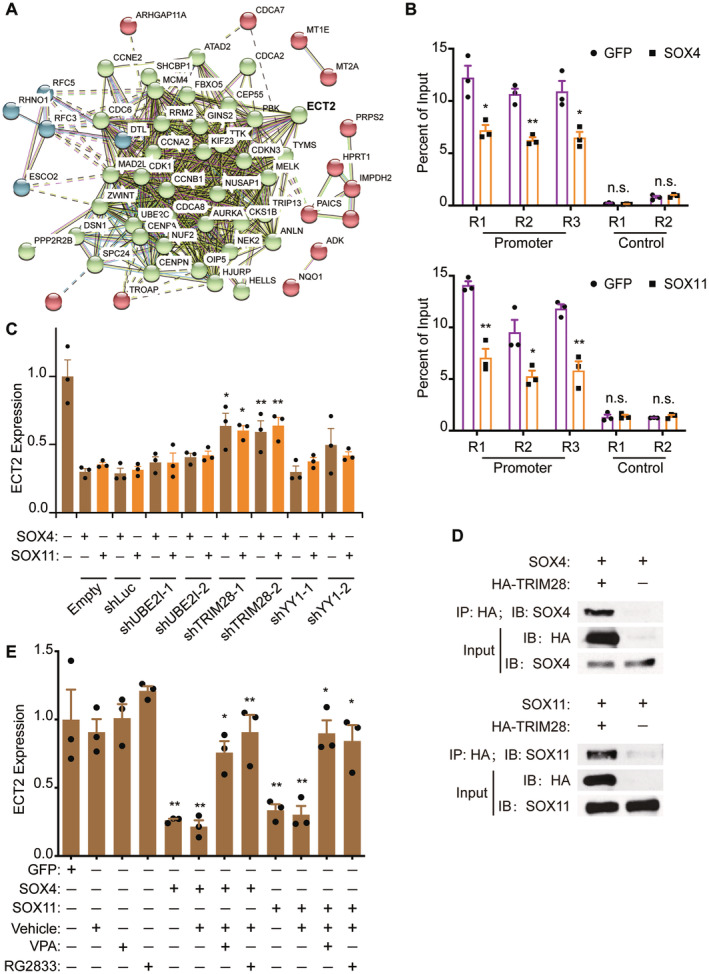
SOXC factors recruit TRIM28 to directly repress ECT2. (A) An analysis of protein–protein interactions is conducted based on RNA‐seq data, with ECT2 identified as a key node within the protein network. (B) ChIP‐qPCR of Histone H3 (acetyl K27) was performed at the ECT2 promoter and control regions in the U251 cell line following the overexpression of SOX4/11, in comparison to the GFP empty vector group (mean ± SEM; *n* = 3; *p < 0.05, **p < 0.01; n.s., not significant; R, Region). (C) qRTPCR analysis was conducted to assess ECT2 expression following the overexpression of SOX4/11 and the knockdown of UBE2I, TRIM28, or YY1 gene expression (mean ± SEM; *n* = 3; *p < 0.05, **p < 0.01). (D) The HA antibody is utilized for the pull‐down of interaction proteins in experiments involving the overexpression of SOX4/11, with or without HA‐TRIM28. Additionally, SOX4 and SOX11 are employed for immunoblotting. (E) qRTPCR analysis was conducted to assess ECT2 expression following the overexpression of SOX4/11 and/or treatment with HDAC inhibitors (VPA and RG2388) (mean ± SEM; *n* = 3; *p < 0.05, **p < 0.01).

Protein–protein interaction analysis, based on RNA‐seq, was conducted to identify the key node of protein, ECT2 (Figure 4A). Either SOX11 or NGN2 were able to downregulate ECT2 gene expression (Figure S7A). We further investigated how SOX4/11 could decrease ECT2 gene expression. In a previous Histone H3 acetyl K27 ChIP‐seq experiment performed on the MRC5 fibroblast cell line, we found that NGN2, both with and without forskolin and dorsomorphin (FD), could reduce the acetylation level of the ECT2 gene at the promoter region (Figure S5A). SOX4/11, critical downstream genes of NGN2, could be induced by NGN2. We hypothesized that NGN2 decreases ECT2 expression due to SOX4/11 overexpression, which could reduce the acetylation level of the ECT2 gene at the promoter region in the U251 cell line. To test this, we performed a ChIP‐qPCR experiment using several pairs of specific primers for the ECT2 promoter region. Compared to the GFP group, SOX4/11 significantly reduced the promoter acetylation level of the ECT2 gene (Figure 4B). Two pairs of primers for the non‐promoter region were used as negative controls and showed no significant change (Figure 4B).

To further elucidate how SOX4/11 could regulate ECT2 gene expression, several universal co‐factors were selected for testing, including TRIM28, YY1, and UBE2I. For each factor, two shRNA constructs co‐expressing mCherry were established, and the knockdown efficiency was confirmed by qRT‐PCR in the U251 cell line (Figure S6A–C). Notably, among these potential co‐factors, the knockdown of TRIM28 could partially rescue the ECT2 expression that was reduced by SOX4/11 (Figure 4C). Importantly, a Co‐IP experiment demonstrated a direct protein–protein interaction between TRIM28 and SOX4/11 (Figure 4D). A recent study showed that TRIM28 regulates the acetylation level by recruiting HDAC [40]. To further confirm that SOX4/11 regulates the acetylation level via TRIM28, specific HDAC chemical inhibitors were employed. Consistently, the inhibition of HDAC by chemical inhibitors (VPA or RG2833) significantly rescued ECT2 gene expression (Figure 4E).

### 2.5 ECT2 Downregulation Mimics SOXC Function in Reprogramming of Glioma Cells

**FIGURE 5:**
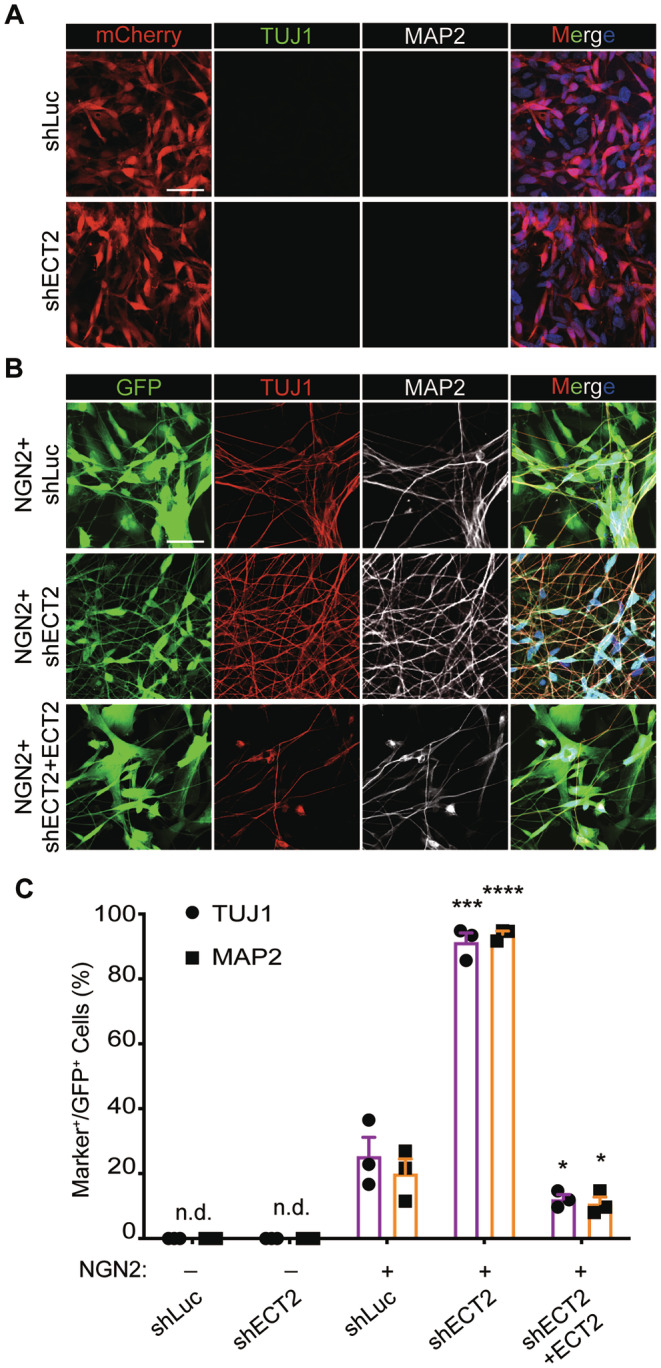
ECT2 downregulation mimics SOXC function in reprogramming of glioma cells. (A) The efficiency of reprogramming has been assessed using immunocytochemistry, employing the neural markers TUJ1 and MAP2. This evaluation was conducted 12 days postinfection with shLuc and shECT2 lentivirus in the U251 cell line. (B) The efficiency of reprogramming has been assessed using immunocytochemistry, employing the neural markers TUJ1 and MAP2. This evaluation was conducted 12 days postinfection with NGN2 + shLuc, NGN2 + shECT2 and NGN2 + shECT2 + ECT2 lentivirus in the U251 cell line. (C) Quantification of Tuj1 and Map2 positive cells, which was normalized to GFP positive cell (mean ± SEM; *n* = 3; *p < 0.05, ***p < 0.001, ****p < 0.0001; n.d., non detectable).

To further validate the role of ECT2 in neuronal reprogramming, we engineered constructs of shECT2, either co‐expressed with NGN2 vectors or independently, as well as an ECT2 overexpression vector. The efficacy of these constructs was confirmed through quantitative Real‐Time PCR (qRTPCR) and western blot assays (Figure S7B–E). Upon examination at 12 days post‐virus infection (dpi), we observed that approximately 20% of NGN2‐expressing U251 cells underwent a dramatic morphological transformation into bipolar or multipolar elongated neuronal forms, robustly expressing TUJ1 and MAP2. The incorporation of ECT2 knockdown significantly promoted this morphological transformation and enhanced the reprogramming efficiency to approximately 90%, as measured by the ratio of cells expressing TUJ1 and MAP2 (Figure 5B,C). Exogenous expression of ECT2 can block the coordinated promotion of NGN2 reprogramming mediated by shECT2 (which knocks down endogenous ECT2 expression through interference with non‐coding sequences) (Figure 5B,C). However, in the absence of the pioneer factor NGN2, ECT2 knockdown failed to induce TUJ1 and MAP2 expression (Figure 5A,C). In summary, our findings highlight the critical role of ECT2 in neuronal reprogramming and establish it as a crucial downstream target gene regulated by SOX4/11.

### 2.6 Differential Regulation of Rho Activities by SOXC Factors During Cell Reprogramming

**FIGURE 6:**
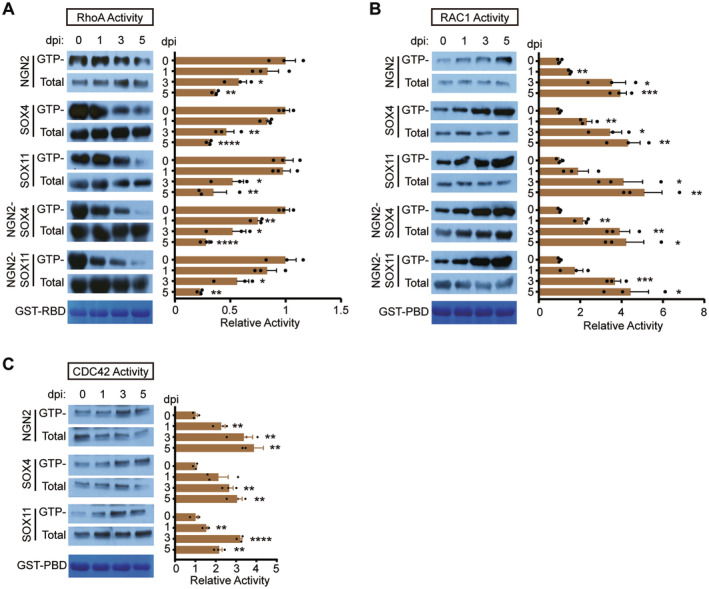
Differential regulation of Rho activities by SOXC factors during cell reprogramming. (A) The GST‐RBD has been employed to precipitate GTP‐RhoA at various time points (0, 1, 3, 5, 7 days) following the infection by different individual lentiviruses (SOX4, SOX11, NGN2, NGN2‐SOX4, and NGN2‐SOX11). The RhoA antibody has been utilized for the detection of both total RhoA and GTP‐RhoA (mean ± SEM; *n* = 3; *p < 0.05, **p < 0.01, ****p < 0.0001). (B) The GST‐PBD has been employed to precipitate GTP‐RAC1 at various time points (0, 1, 3, 5, 7 days) following the infection by different individual lentiviruses (SOX4, SOX11, NGN2, NGN2‐SOX4, and NGN2‐SOX11). RAC1 antibodies have been used for detecting total RAC1 and GTP‐RAC1 (mean ± SEM; *n* = 3; *p < 0.05, **p < 0.01, ***p < 0.001). (C) The GST‐PBD has been employed to precipitate GTP‐CDC42 at various time points (0, 1, 3, 5, 7 days) following the infection by different individual lentiviruses (SOX4, SOX11, and NGN2). CDC42 antibodies have been used for detecting total CDC42 and GTP‐CDC42 (mean ± SEM; *n* = 3; **p < 0.01, ****p < 0.0001).

Recent studies have established ECT2 as a critical upstream regulator of RhoA, with the reduction of ECT2 leading to a decrease in RhoA activity [41]. Additionally, signal crosstalk has been observed between RhoA and RAC1/CDC42, both of which play a crucial role in axon‐outgrowth [42]. To further investigate the activity of RhoA and RAC1/CDC42 during reprogramming, we conducted GST pull‐down experiments. Initially, GST‐RBD was utilized to isolate GTP‐RhoA at various time points (0‐, 1‐, 3‐, and 5‐days postinfection). Our results demonstrated that SOX4/11 could significantly reduce GTP‐RhoA levels without affecting total RhoA (Figure 6A). Consistently, NGN2, NGN2‐SOX4, and NGN2‐SOX11 were also found to inhibit RhoA activity. In parallel, agarose beads containing GST‐PBD were produced and used for the isolation of RAC1/CDC42 at the same time points. Immune blotting results for RAC1/CDC42 revealed that SOX4/11 and/or NGN2 could dramatically induce the production of GTP‐RAC1/CDC42 (Figure 6B,C). In summary, our findings indicate that SOX4/11 and/or NGN2 can influence the activity of RhoA and RAC1/CDC42, suggesting their critical role in the reprogramming process.

### 2.7 Small Molecules Targeting RhoA and Its Effectors

**FIGURE 7:**
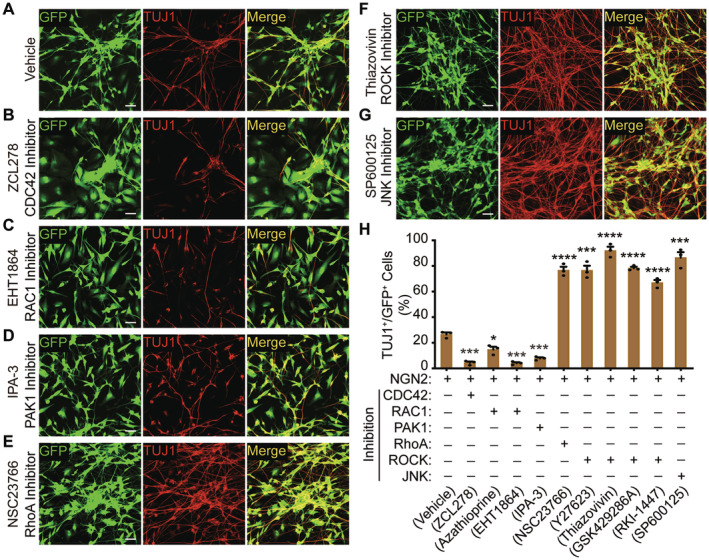
Small molecules that target RhoA and its effectors influence the efficiency of reprogramming mediated by NGN2. (A) Immunocytochemistry staining of TUJ1 in the U251 cell line following NGN2 lentivirus infection, with treatment of 0.1% DMSO at 12 dpi serving as the vehicle control group. (B) Immunocytochemistry staining of TUJ1 in the U251 cell line following NGN2 lentivirus infection, with treatment of CDC42 inhibitor (ZCL278) at 12 dpi. (C) Immunocytochemistry staining of TUJ1 in the U251 cell line following NGN2 lentivirus infection, with treatment of RAC1 inhibitor (EHT1864) at 12 dpi. (D) Immunocytochemistry staining of TUJ1 in the U251 cell line following NGN2 lentivirus infection, with treatment of PKA inhibitor (IPA‐3) at 12 dpi. (E) Immunocytochemistry staining of TUJ1 in the U251 cell line following NGN2 lentivirus infection, with treatment of RhoA inhibitor (NSC 23766) at 12 dpi. (F) Immunocytochemistry staining of TUJ1 in the U251 cell line following NGN2 lentivirus infection, with treatment of ROCK inhibitor (Thiazovivin) at 12 dpi. (G) Immunocytochemistry staining of TUJ1 in the U251 cell line following NGN2 lentivirus infection, with treatment of JNK inhibitor (SP600125) at 12 dpi. (H) Quantification of TUJ1 positive cells, which was normalized to GFP positive cell (mean ± SEM; *n* = 3; *p < 0.05, ***p < 0.001, ****p < 0.0001).

To ascertain the roles of RhoA and RAC1/CDC42 in the reprogramming process, we employed a series of chemicals associated with the RhoA and RAC1/CDC42 signaling pathways. The reprogramming efficiency of U251 was evaluated at 12‐days postinfection (dpi) using Tuj1 staining, and the results were normalized to GFP‐positive cells. Our findings indicate that treatment with the CDC42 inhibitor (ZCL278) resulted in a significant decrease in reprogramming efficiency (Figure 7A,B,H). Similarly, RAC1 inhibitors (EHT1864) also impeded the NGN2‐mediated reprogramming of U251 cells into neuron cells (Figure 7A,C,H). Furthermore, when the PKA inhibitor (IPA‐3), a downstream target of RAC1/CDC42, was introduced during the reprogramming process, we observed a decrease in reprogramming efficiency (Figure 7A,D,H). In contrast, the RhoA inhibitor (NSC 23766) enhanced NEUROG2‐mediated reprogramming of U251 cells into neuron cells (Figure 7A,E,H). Likewise, ROCK inhibitors (Thiazovivin) also improved the reprogramming efficiency mediated by NGN2 (Figure 7A,F,H). Additionally, the JNK inhibitor (SP600125), a downstream factor of ROCK, promoted NGN2‐mediated reprogramming of U251 cells into neuron cells (Figure 7A,G,H). In summary, our findings underscore the critical role of the RhoA/ROCK and RAC1/CDC42 pathway axes in neuronal reprogramming derived from the U251 cell line.

## 3 Discussion

In our preceding study, we elucidated the capability of NGN2 and SOX11 to transform glioblastoma cells into neurons, resulting in cell cycle arrest [3]. The current research augments this understanding by confirming the pivotal role of SOXC factors in facilitating NGN2‐induced fate reprogramming of human glioblastoma cells. We pinpointed ECT2 as a crucial target gene under the regulation of SOX4/11. The mechanism unveiled involves SOXC factors enlisting TRIM28 to directly inhibit ECT2 expression by deacetylating its promoter region. The inhibition of RhoA/ROCK pathway enhances the ability of NGN2 to reprogram U251 cells into neurons, whereas blocking the RAC1/CDC42 pathway impedes NGN2's reprogramming ability in the U251 cell line. Collectively, our systematic approach has allowed us to identify the underlying molecular interactions during neuron reprogramming derived from glioblastoma, providing a potential new direction for drug discovery in glioblastoma therapy.

Pro‐neural factors have been demonstrated to induce tumor‐initiating cells to go through neuronal differentiation to inhibit tumorigenesis, which has been thought as a novel therapeutic strategy for glioblastoma [4, 6, 43]. In our study, human SOX4 and SOX11 either could highly efficient (95%) reprogramming U251 cell into neuron cell, which is a promising strategy for glioblastoma therapy. Both SOX4 and SOX11 belong to the SOXC family, which plays critical role in many different tissues, including nervous system development. A recent study showed that HMG‐box transcription factors Sox4 and Sox11 are of critical importance, downstream from pro‐neural bHLH proteins, for the establishment of pan‐neuronal protein expression [14]. The underlying mechanism of reprogramming is critical to translate this technology for clinical application.

The dynamic changes in the transcriptome are instrumental in identifying potential signaling pathways or critical genes during neuron reprogramming, facilitated by transcription factors such as NGN2 and SOX11. Our comprehensive analysis reveals a significant upregulation of genes associated with neurogenesis. These augmented genes predominantly contribute to axon guidance, nervous system development, and synaptic vesicle endocytosis. Conversely, genes associated with mitotic nuclear division, cell division, and nucleosome assembly are markedly downregulated. This shift in the transcriptomic landscape aligns with the ongoing neuron reprogramming process. Both NGN2 and SOX11 have been empirically validated to induce the expression of neuron pattern genes essential for neurogenesis [44, 45, 46, 47, 48]. Given their analogous roles in neurogenesis, we observed a substantial overlap between upregulated and downregulated genes within both NGN2 and SOX11 groups. Upon scrutinizing the RNA‐seq data, we found that the overexpression of NGN2 could induce the expression of SOX4 and SOX11. Furthermore, NGN2 also has the capacity to regulate the expression of the SOX4 gene in the MRC5 cell line [10]. This is corroborated by our ChIP‐seq experiment, which revealed that NGN2 could target the SOX4/11 genes. In U251 cells where SOX4 and SOX11 have been completely deleted (double knockout, or dKO), the expression of TUJ1 is significantly reduced, and these cells fail to exhibit neuron‐like morphology during NGN2‐mediated fate reprogramming. Consequently, our research underscores the indispensable role of SOXC factors in the NGN2‐mediated fate reprogramming of human glioblastoma cells.

ECT2 has been identified as one of the key node gene by protein–protein interaction analysis of RNA‐seq. Knockdown ECT2 could significantly enhance NGN2 reprogramming U251 cell into neuron cell. In reverse, overexpression of ECT2 could block NGN2 reprogramming U251 cell into neuron. In summary, we found that downregulation of ECT2 is critical for neuron reprogramming. NGN2 and SOX11 both have been shown to significantly decrease ECT2 gene expression. Subsequently, we explored how SOX4/11 could regulate ECT2 gene expression.

Ectopic SOX4 expression reduced endogenous PU.1 mRNA levels in HL60 promyelocytes, and decreased Sfpi1 mRNA levels were also observed in the spleens of leukemic and preleukemic mice receiving Sox4 virus‐infected wild‐type bone marrow cells. In addition, Sox4 protein bound to a critical upstream regulatory element of Sfpi1 in ChIP assays [49]. In our study, we found knockdown TRIM28 will partly rescue ECT2 gene expression. In addition, the acylation level of promotor region of ECT2 can be reduced by SOX4 or SOX11 overexpression. Recently, TRIM28 has been shown to influence chromatin acylation level by recruiting HDAC1 [40]. Consistently, we found SOX4/11 could directly interact with TRIM28 and inhibit HDAC1 by small molecules, which could also partly rescue ECT2 gene expression. In summary, in our study we illustrate the detailed mechanism of how SOX4/11 could regulate ECT2 gene expression by recruiting TRIM28 and HDAC1.

It is well established that ECT2 is directly associated with the RhoA/ROCK pathway [41, 50, 51]. This association enables ECT2 to promote the activation of the RhoA/ROCK pathway. Furthermore, existing research indicates a crosstalk between RhoA and RAC1/CDC42 [52, 53]. The equilibrium between the RhoA and RAC1/CDC42 pathways influences GTPase cascades, which play a pivotal role in morphological regulation and cytoskeleton organization through the formation of stress fibers, lamellipodia, and filopodia, respectively. Therefore, it can be inferred that the function of ECT2 in neuron reprogramming may be attributed to its modulation of RhoA/ROCK and RAC1/CDC42 activity. In our study, we discovered that SOX4, SOX11, or NGN2 could augment the activation of RAC1/CDC42 and attenuate RhoA activity. Previous studies have shown that the blockade of the Rho/ROCK pathway can enhance axon regeneration [54]. Moreover, the activation of CDC42 can increase growth cone motility and filopodia formation, which are crucial for neuron axon outgrowth [55]. Additionally, it has been demonstrated that the inhibition of Rho can enhance axon outgrowth of precerebellar neurons, whereas the inhibition of RAC and CDC42 can impair neurite outgrowth [42]. When specific chemical compounds were applied to interrupt different signal pathways in our study, we found that a RhoA/ROCK inhibitor could enhance the reprogramming of U251 into neurons by NGN2, whereas the blockade of RAC1/CDC42 could inhibit the neuron reprogramming process. Consistently, a recent study demonstrated that SOX11 is required for neuron cell survival and axon outgrowth [56], which may also be partly attributed to this signal pathway. In summary, ETC2 can directly regulate RhoA activity. RhoA/ROCK and RAC1/CDC42 pathways play critical roles in axon growth.

In conclusion, our study has empirically demonstrated that NGN2 and SOX4/11 can reprogram glioblastoma cells into neurons with high efficiency. We have elucidated a stepwise mechanism underlying this reprogramming process. Our findings underscore the pivotal roles of the RhoA/ROCK and RAC1/CDC42 pathways in neuron reprogramming. These insights pave the way for innovative directions in glioblastoma therapy. Interestingly, our study suggests that the use of specific chemicals, rather than transcription factors, could be sufficient to inhibit glioblastoma growth through cell reprogramming. This discovery could potentially revolutionize the current therapeutic strategies for glioblastoma.

## 4 Materials and Methods

### 4.1 Cell Culture

HEK293 (CRL‐1573, ATCC) and U251 human glioma cells (human U‐251 MG glioblastoma cell line, Sigma) were maintained in Dulbecco's Modified Eagles Medium (DMEM; GE Healthcare Life Science) supplemented with 10% fetal bovine serum and 1% penicillin–streptomycin (Thermo Fisher). Cells were passaged every 2–3 days before confluency. For virus‐transduced U251 cells on Matrigel‐coated coverslips in 24‐well plates, culture medium was switched 48 h after viral transduction to reprogramming medium consisting of DMEM/F12/neurobasal (2:2:1 dilutions), 0.8% N2/0.4% B27, 10 μM forskolin (Sigma), and 1 μM dorsomorphin (Millipore). Wherever indicated, small molecules from Selleck Chemicals or Sigma were also included in the medium. A list of small molecules was included in Table S2. All cells were maintained at 37°C in a humidified incubator with 5% CO_2_.

### 4.2 Plasmids and Lentivirus Production

The third generation CS‐CDF‐CG‐PRE lentiviral vector was used for expression of GFP, HA‐NGN2‐IRES‐GFP, HA‐SOX11‐IRES‐GFP, HA‐NGN2‐IRES‐GFP‐T2A‐SOX4, HA‐NGN2‐IRES‐GFP‐T2A‐SOX11, HA‐ECT2‐IRES‐GFP, mCherry‐shLuc, mCherry‐shECT2, mCherry‐shUBE2I, mCherry‐shTRIM28, mCherry‐shYY1, NGN2‐IRES‐GFP‐shLuc, and NGN2‐IRES‐GFP‐shECT2. The lentiCRISPRv2 vector (Addgene plasmid # 52,961; http://n2t.net/addgene:52961; RRID: Addgene_52,961) was used to express sgRNAs. For some experiments, the selection marker puromycin was replaced with mCherry for the identification of virus‐transduced cells. All plasmids were sequenced for confirmation. VSV‐G‐pseudotyped lentivirus was prepared essentially as previously described [9, 57]. Briefly, lentiviral vectors and packaging plasmids (pMDL, VSV‐G, and pREV) were co‐transfected into HEK293 cells. Sixteen hours later, culture medium was refreshed with growth medium. Virus‐containing media were then collected, filtered, and stored at 4°C.

### 4.3 Immunocytochemistry and Cell Proliferation

U251 cells on Matrigel‐coated coverslips in a 24‐well plate were twice washed with ice‐cold PBS and fixed with 4% PFA for 15 min at RT. After permeabilization with two washes of PBST (1 × PBS containing 0.2% Triton X‐100) for 5 min each at RT, cells were then blocked with 3% BSA‐containing PBST solution and sequentially incubated with primary and secondary antibodies. The following primary antibodies were used in blocking solution: TUJ1 (rabbit, Covance PRB‐435P, 1:1000; chicken, Aves TUJ‐S, 1:1000), MAP2 (mouse, Sigma M4403, 1:1000), Ki67 (rabbit, Abcam ab15580, 1:1000), Brdu (rat, Accurate Chemical YSRTMCA2060GA, 1:500), and NeuN (rabbit, Abcam ab177487, 1:1000). The corresponding Alexa Fluor 488‐, 594‐, or 647‐conjugated secondary antibodies (Jackson Laboratory, 1:500) were also used. Nuclei were counterstained with Hoechst 33342 (1 μg/mL in PBST). Cells were imaged by using a Zeiss LSM 700 confocal microscope. For quantifications, cells were processed in triplicates and counted in 3 random areas for each replicate. Cell growth and proliferation were also measured by using the ATP‐dependent CellTiter‐Glo 2.0 Luminescent Assay kit (Promega). Briefly, cells were seeded in 96‐well plates (1000 cells per well) and were then transduced with the indicated lentivirus 16 h later. Six wells were used for each condition. Cells were monitored by the coexpressed GFP marker to confirm high transduction efficiency (∼95%). ATP‐dependent luminescence for each well was measured by using a plate reader (BioTek) and was then normalized to the control GFP at 3 dpi.

### 4.4 Fluorescence Activated Cell Sorting (FACS)

After a gentle wash with ice‐cold PBS, cells in a 10‐cm dish were treated with Accutase for 3–4 min at RT and collected by centrifugation at 1800 rpm for 3 min. Cell pellets were washed with cold PBS and re‐centrifugation and finally resuspended at a centration of 5 × 10^6^/mL in PBS containing 5 mM HEPES (pH 7.0) and 5 mM EDTA. GFP‐positive cells were collected by using a MoFlo Cytometer (Beckman Coulter).

### 4.5 Total RNA Isolation and RNA‐Sequencing (RNA‐Seq)

As previously described [10], high quality RNAs were isolated from sorted cells by using the TRIzol reagents (Life Technologies) and used for cDNA library preparations by using an Illumina kit. Single‐end 50‐base length sequences were generated on an Illumina HiSeq 2500 System and mapped to GRCh37/hg19. Genes were defined as significant when the following three criteria were met: Student's *t*‐test *p* value ≤ 0.05, at least 2‐fold gene expression change relative to the GFP control, and the triplicate average of ln (FPKM) ≥ 1 in either the experimental condition or control condition. Gene ontology analysis was performed with the DAVID Functional Annotation Tool (version 6.7). Gene expression heat map was drawn by MeV software package.

### 4.6 Chromatin Immunoprecipitation‐Sequencing (ChIP‐Seq)

This was performed essentially as previously described [10]. Briefly, a total of ~1.6 × 10^7^ cells were processed for formaldehyde‐crosslinking and isolation of fragmented chromatin. Approximately 150 μg of chromatin was used for immunoprecipitation with 5 μg HA antibody (Abcam, AB9110) or antibody against histone H3 acetyl K27 (Abcam, ab4729). The immunoprecipitated chromatin was washed, reverse‐crosslinked, and purified for genomic DNA. Ten ng each of input and immunoprecipitated DNA were used to build libraries by using the NEBNext ChIP‐Seq Library Prep Master Mix Set for Illumina kit (New England Biolabs, E6240S). Biological triplicate libraries were generated from independent samples. Single‐end 50‐base sequence reads were generated by an Illumina HiSeq 2500 System and aligned to GRCh37/hg19. HOMER was used for the following analysis including Peak calling, annotation, motif discovery, and GO analysis. The UCSC Genome Browser was used for track visualization.

### 4.7 Chromatin Immunoprecipitation‐Quantitative PCR (ChIP‐qPCR)

After chromatin immunoprecipitation with an antibody against histone H3 acetyl K27, purified genomic DNA was determined by using the FastStart Universal SYBR Green Master Mix (ROX) (Roche, 04913922001) on a 7900HT Fast Real‐Time PCR System (Applied Biosystems, 4,329,002). Three primer pairs were surrounding the ECT2 promoter region, and two control primer pairs were in the intergenic region about 9 kb upstream of the ECT2 gene. Primer sequences are included in Table S3.

### 4.8 Quantitative RT‐PCR (qRT‐PCR)

Total RNA was isolated by using the TRIzol reagents (Life Technologies). Reserve transcription was performed by using 1 μg purified total RNA and SuperScript III Reverse Transcriptase (Invitrogen, 18080–093) with random primers. Amplification was performed on 7900HT Fast Real‐Time PCR System (AppliedBiosystems, 4329002) by using FastStart Universal SYBR Green Master Mix (ROX) (Roche, 04913922001). Amplification specificity was confirmed by dissociation curves. Primer sequences are listed in Table S1.

### 4.9 Gene Knockouts and Knockdowns

The CRISPR/Cas9 system was used for generation of gene knockout U251 cells. The target oligo sequences for sgRNAs were selected based on CRISPR design tool (http://crispr.mit.edu/) and cloned into the lentiCRISPR v2 vector. After viral transduction and puromycin selection (0.4 μg/mL), cell colonies were screened by sequencing the PCR‐amplified genomic DNAs surrounding the sgRNA‐target sites. A second round of viral transduction and screens was conducted to select cells with double knockouts. The microRNA‐based short hairpin RNAs (shRNAs) according to SplashRNA and miR‐E designs were used for all gene knockdown experiments [58]. Briefly, DNA oligo sequences were subcloned into the miR‐E backbone that was linked to either GFP or mCherry. Knockdown efficiency was determined by qRT‐PCR by using gene‐specific primer pairs. All primer/oligo sequences are listed in Table S3.

### 4.10 Protein Co‐Immunoprecipitation and Western Blotting

U251 cells in a 10‐cm dish were transfected with plasmids by using the Lipofectamine 2000 reagents (Thermo Fischer). Three days later, whole cell lysates were collected by sonication in 500 μL lysis buffer consisting of 1xPBS, 1 mM EDTA, 0.5% (v/v) Triton X‐100, and 1% (v/v) complete EDTA‐free Protease Inhibitor Cocktail (Roche). Cell lysates were cleared by centrifugation at 14,000 rpm for 10 min at 4°C. With 10% of the lysates saved as input, the remaining was precleared through incubation with 25 μL Magnetic Protein G Dynabeads for 1 h at 4°C. After a brief centrifugation, the supernatant was transferred to a new tube and incubated with 25 μL anti‐HA Magnetic Beads (Thermo Fischer) for 2 h with gentle rotation at 4°C. Beads were magnetically collected and washed with lysis buffer for 4 times at 4°C. After the final wash, beads were resuspended in 50 μL 1 × protein loading buffer and heated for 5 min with shaking at 70°C. After boiling input samples in 1 × protein loading buffer (60 mM Tris/HCl, pH 6.8, 2% SDS, 1.25% 2‐mercaptoethanol, 10% glycerol, and 0.01% bromophenol blue), both inputs and immunoprecipitates were separated on a 10% SDS‐PAGE gel. Proteins were transferred onto a PVDF membrane and blocked with 5% milk‐containing PBST solution for 1 h at RT. The membrane was then sequentially incubated with specific primary and HRP‐conjugated secondary antibodies and washed with PBST solution. Protein bands were visualized with the ECL system (Thermo Fischer) and analyzed by Image J software.

### 4.11 Rho Activity Assays

The pGEX‐2 T‐GST‐RBD and pGEXTK‐Pak1 70–117 plasmids were gifts of Martin Schwartz (Addgene plasmid # 15247) [59] and Jonathan Chernoff (Addgene plasmid # 12217), respectively. According to a standard protocol [60], GST‐RBD and GST‐Pak1 70–117 (GST‐PBD) fusion proteins were purified from BL21 (DE3) *E. coli*. U251 cells in a 10‐cm dish were collected at the indicated time points after viral transduction and lysed by sonication in 300 μL lysis buffer (50 mM Tris, pH 7.6, 150 mM NaCl, 1% Triton X‐100, 20 mM MgCl_2_ and 1% protease inhibitor cocktail). Cell lysates were cleared through centrifugation at 12,000 rpm for 10 min at 4°C and diluted to 1 μg/mL in lysis buffer. Approximately 800 μg cell lysates were incubated with 60 μg GST‐RBD or GST‐PBD beads for 1 h at 4°C. Beads were then washed 3 times with lysis buffer and resuspended in 60 μL 1 × protein loading buffer. After boiling for 5 min, both input and GST pull‐down samples were separated on a 10% SDS‐PAGE gel and processed for western blotting with specific antibodies. The following primary antibodies were used: CDC42 (Rabbit, Proteintech, 10,155‐1‐AP, 1:1000), RAC1 (Rabbit, Proteintech, 24,072‐1‐AP, 1:1000), RhoA (Rabbit, Proteintech, 10,749‐1‐AP, 1:1000). GST‐RBD and GST‐PBD were also stained with Coomassie brilliant blue as loading controls.

### 4.12 Statistics

Experiments were performed in triplicates unless otherwise indicated. Normality test for data distribution assession were performed using GraphPad Prism 9. Data are presented as mean ± SEM. Statistical analysis was performed by homoscedastic two‐tailed Student's *t*‐test. Significant differences are indicated by *p < 0.05, **p < 0.01, ***p < 0.001, or ****p < 0.0001.

### 4.13 Accession Numbers

The next‐generation sequencing data has been stored in the NCBI database, with the accession numbers being GSE112648 (RNA‐seq) and GSE112645 (ChIP‐seq).

## Author Contributions

**Jianjing Yang**, **Chun‐Li Zhang**, and **Qichuan Zhuge** conceived and designed the experiments. **Jianjing Yang** carried out most of the experiments and data analysis. **Xiaohong Zhu**, **Fan Wang**, **Zhen Chen**, **Ying Zhang**, **Jiawei Chen**, and **Haoqi Ni** carried out part of experiments and provided critical technical assistance. **Jianjing Yang** and **Chun‐Li Zhang** wrote and edited the manuscript. All authors gave feedback and agreed on the final version of the manuscript.

## Conflicts of Interest

The authors declare no conflicts of interest.

## Supporting information


**FIGURES1** NGN2 and SOXC reprogram U251 cell into mature neuron.
(A, B) The reprogramming efficiency of mature neuron has been assessed using immunocytochemistry, employing the neural markers NeuN and MAP2. This evaluation was conducted 21 days postinfection with GFP, GFP‐NGN2‐SOX4, and GFP‐NGN2‐SOX11 lentivirus in the U251 cell line.
(C) Quantification of NeuN positive cells, which were normalized to GFP positive cell (mean ± SEM; *n* = 3).

**FIGURES2** NGN2 and SOXC reprogram primary glioblastoma cell (GBM) into neuron.
(A) Schematic diagram illustrates culture process of reprogramming glioblastoma cell into neuron.
(B) Representative image of Tuj1 and Map2 staining at 12 days after GFP‐NGN2‐SOX4 virus infection in GBM cell (Scale bar = 50 μm).
(C) Representative image of Tuj1 and Map2 staining at 12 days after GFP‐NGN2‐SOX11 virus infection in GBM cell (Scale bar = 50 μm).
(D) Quantification of Tuj1 and Map2 positive cells, which were normalized to GFP positive cell at 12 days after virus infection in GBM cell (mean ± SEM; *n* = 3 per group).

**FIGURES3** The role of SOXC in NGN2‐mediated reprogramming.
(A) The NGN2 binding track of SOX4 and SOX11 genes.
(B) The mRNA expression levels of SOX4 and SOX11 were examined using RNA‐seq analysis (*n* = 3).
(C) Western Blot analysis has been employed to determine the expression levels of SOX4/11 proteins following the overexpression of NGN2 in the U251 cell line at 3 dpi.
(D) Cell morphology change of U251 after infection of GFP‐NGN2 lentivirus at 12 dpi with or without SOX4/11 double knockout (Scale bar = 400 μm).
(E) Western Blot confirming SOX4/11 knockout cell line establishment.

**FIGURES4** NGN2 and SOX11 potently inhibit glioblastoma cell proliferation and growth.
(A) Immunocytochemistry of proliferating glioblastoma cells indicated by Ki67‐staining. Virus‐transduced U251 glioblastoma cells are indicated by the coexpressed GFP (Scale bar = 50 μm).
(B, C) Confocal images of glioblastoma cells undergoing DNA replication. BrdU was applied 2 h before immunocytochemistry. GFP expression indicates virus‐transduced U251 glioblastoma cells (Scale bar = 50 μm).
(D) Quantification of Ki67+ glioblastoma cells at the indicated time points (mean ± SEM; *n* = 3; ***p < 0.001).
(E, F) Quantification of BrdU+ glioblastoma cells at the indicated time points (mean ± SEM; *n* = 3; ***p < 0.001).
(G) A time‐course analysis of relative cell proliferation by measuring ATP‐dependent luminescence (mean ± SEM; *n* = 6; *p < 0.05 and ***p < 0.001).

**FIGURES5** NGN2 can reduce the acetylation level of the ect2 gene at the promoter region.
Histone h3 acetyl k27 chip‐seq in mrc5 cell line at 3 dpi with or without ngn2 infection (3 *n*, 3 days postinfection of NGN2).

**FIGURES6** Knockdown the mRNA expression level of UBE2I, RIM28, and YY1 by shRNA.
(A) qRT‐PCR analysis for shUBE2I knockdown efficiency at 3 dpi in U251 cell line (mean ± SEM; *n* = 3; **p < 0.01 ***p < 0.001).
(B) qRT‐PCR analysis for shTRIM28 knockdown efficiency at 3 dpi in U251 cell line (mean ± SEM; *n* = 3; *p < 0.05 **p < 0.01).
(C) qRT‐PCR analysis for shYY1 knockdown efficiency at 3 dpi in U251 cell line (mean ± SEM; *n* = 3; *p < 0.05 **p < 0.01).

**FIGURES7** Confirmation of ECT2 Expression.
(A) RNA‐seq data of ECT2 mRNA expression levels in different groups (*n* = 3).
(B) qRT‐PCR analysis for sh_ECT2 knockdown efficiency at 3 dpi in u251 cell line (mean ± SEM; *n* = 3; *p < 0.05).
(C) Western blot analysis for shECT2_1 knockdown efficiency.
(D) qRT‐PCR analysis for NGN2‐shECT2 knockdown efficiency at 3 dpi in u251 cell line (mean ± SEM; *n* = 3; *p < 0.05).
(E) Western blot analysis for NGN2‐shECT2_2 knockdown efficiency and ECT2 overexpression.

**TABLES1** A comprehensive list of differentially expressed genes obtained from RNA‐seq, used for heatmap plotting.

**TABLES2** All primer sequences utilized in the research.

**TABLES3** Information on small molecule compounds used to block various signaling pathways.

## Data Availability

The data that support the findings of this study are available on request from the corresponding author. The data are not publicly available due to privacy or ethical restrictions.
