# Supplementary figures and images for: SOXC Enhances NGN2‐Mediated Reprogramming of Glioblastoma Cells Into Neuron‐Like Cells by Modulating RhoA and RAC1/CDC42 Pathway Activity
Source: CNS Neurosci Ther. 2024 Oct 10;30(10):e70075. doi: 10.1111/cns.70075 (PMC11467166; doi:10.1111/cns.70075)

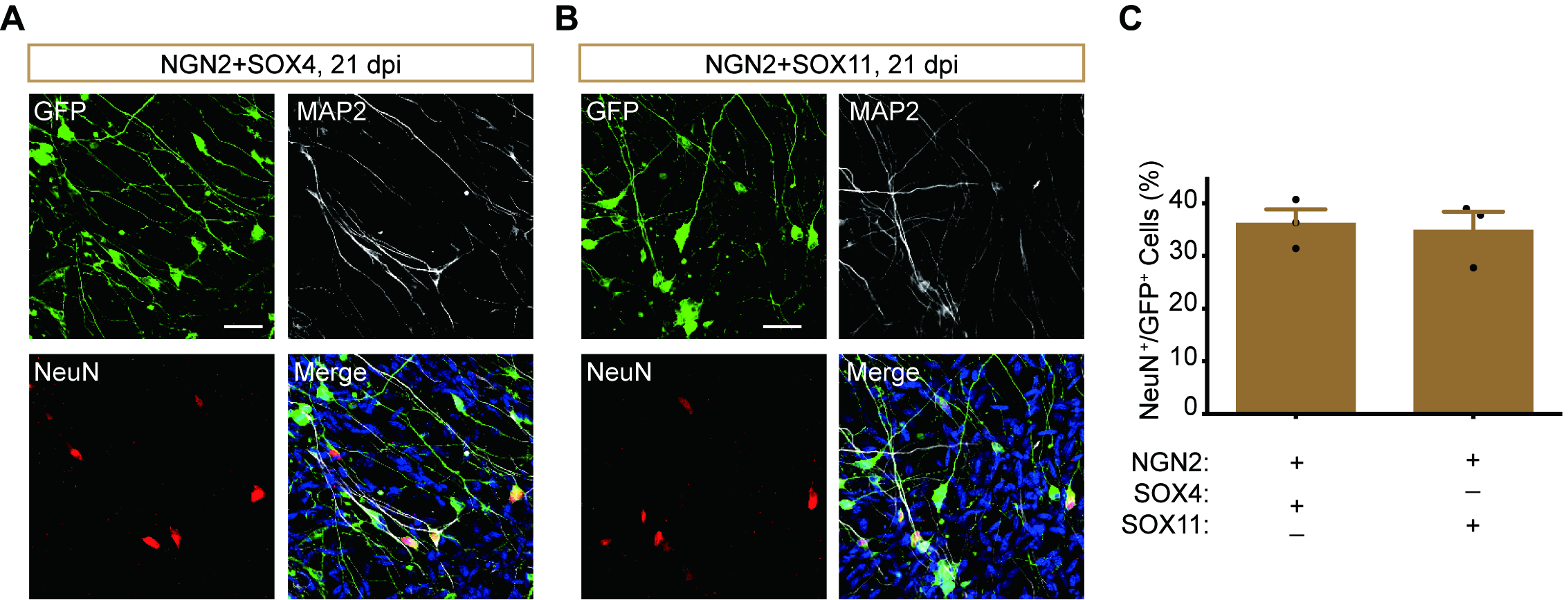

Supplement: Supplementary file 1 — FIGURES1 NGN2 and SOXC reprogram U251 cell into mature neuron. (A, B) The reprogramming efficiency of mature neuron has been assessed using immunocytochemistry, employing the neural markers NeuN and MAP2. This evaluation was conducted 21 days postinfection with GFP, GFP‐NGN2‐SOX4, and GFP‐NGN2‐SOX11 lentivirus in the U251 cell line. (C) Quantification of NeuN positive cells, which were normalized to GFP positive cell (mean ± SEM; n = 3). [file CNS-30-e70075-s007.tif]

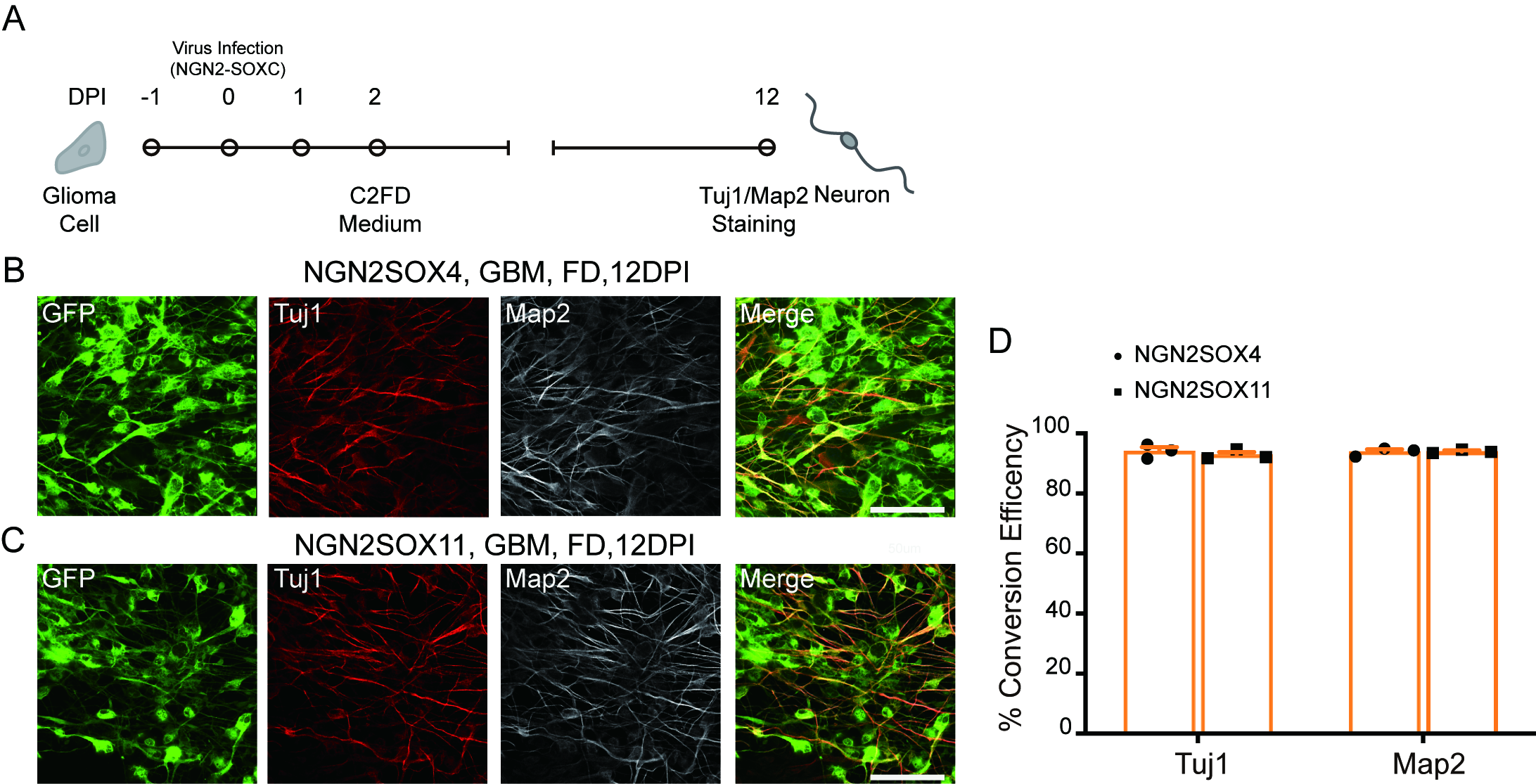

Supplement: Supplementary file 2 — FIGURES2 NGN2 and SOXC reprogram primary glioblastoma cell (GBM) into neuron. (A) Schematic diagram illustrates culture process of reprogramming glioblastoma cell into neuron. (B) Representative image of Tuj1 and Map2 staining at 12 days after GFP‐NGN2‐SOX4 virus infection in GBM cell (Scale bar = 50 μm). (C) Representative image of Tuj1 and Map2 staining at 12 days after GFP‐NGN2‐SOX11 virus infection in GBM cell (Scale bar = 50 μm). (D) Quantification of Tuj1 and Map2 positive cells, which were normalized to GFP positive cell at 12 days after virus infection in GBM cell (mean ± SEM; n = 3 per group). [file CNS-30-e70075-s008.tif]

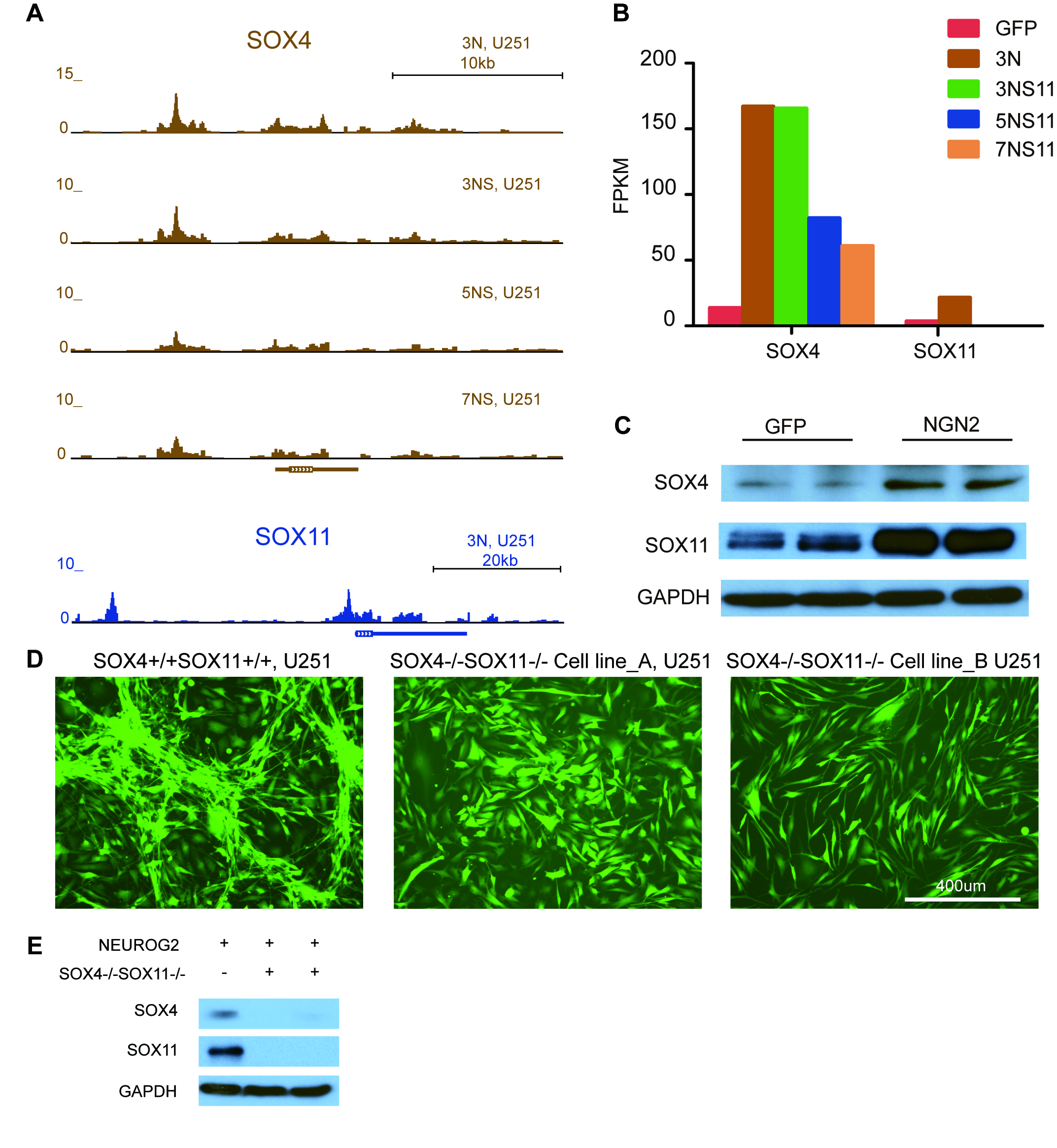

Supplement: Supplementary file 3 — FIGURES3 The role of SOXC in NGN2‐mediated reprogramming. (A) The NGN2 binding track of SOX4 and SOX11 genes. (B) The mRNA expression levels of SOX4 and SOX11 were examined using RNA‐seq analysis (n = 3). (C) Western Blot analysis has been employed to determine the expression levels of SOX4/11 proteins following the overexpression of NGN2 in the U251 cell line at 3 dpi. (D) Cell morphology change of U251 after infection of GFP‐NGN2 lentivirus at 12 dpi with or without SOX4/11 double knockout (Scale bar = 400 μm). (E) Western Blot confirming SOX4/11 knockout cell line establishment. [file CNS-30-e70075-s002.tif]

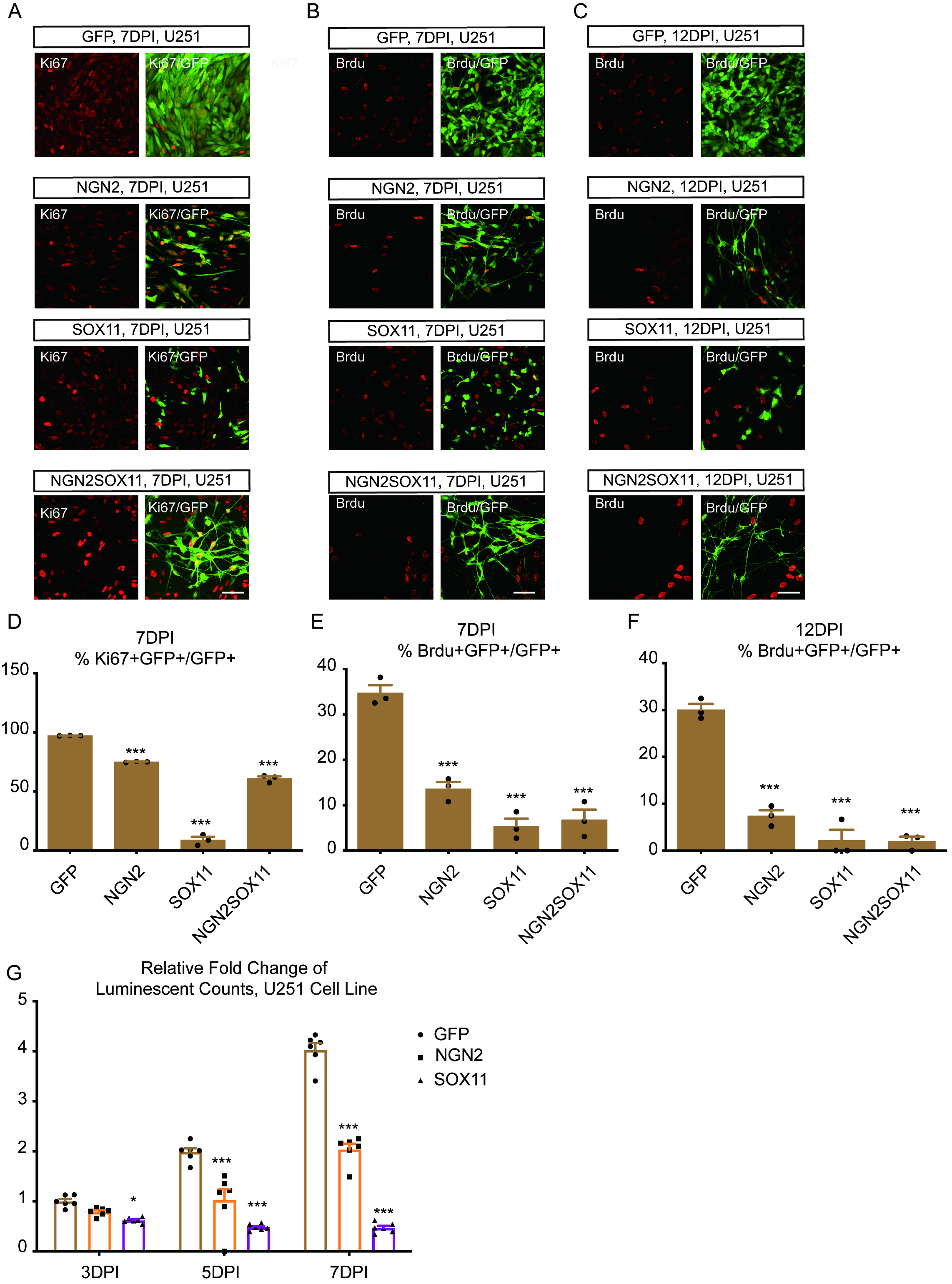

Supplement: Supplementary file 4 — FIGURES4 NGN2 and SOX11 potently inhibit glioblastoma cell proliferation and growth. (A) Immunocytochemistry of proliferating glioblastoma cells indicated by Ki67‐staining. Virus‐transduced U251 glioblastoma cells are indicated by the coexpressed GFP (Scale bar = 50 μm). (B, C) Confocal images of glioblastoma cells undergoing DNA replication. BrdU was applied 2 h before immunocytochemistry. GFP expression indicates virus‐transduced U251 glioblastoma cells (Scale bar = 50 μm). (D) Quantification of Ki67+ glioblastoma cells at the indicated time points (mean ± SEM; n = 3; ***p < 0.001). (E, F) Quantification of BrdU+ glioblastoma cells at the indicated time points (mean ± SEM; n = 3; ***p < 0.001). (G) A time‐course analysis of relative cell proliferation by measuring ATP‐dependent luminescence (mean ± SEM; n = 6; *p < 0.05 and ***p < 0.001). [file CNS-30-e70075-s010.tif]

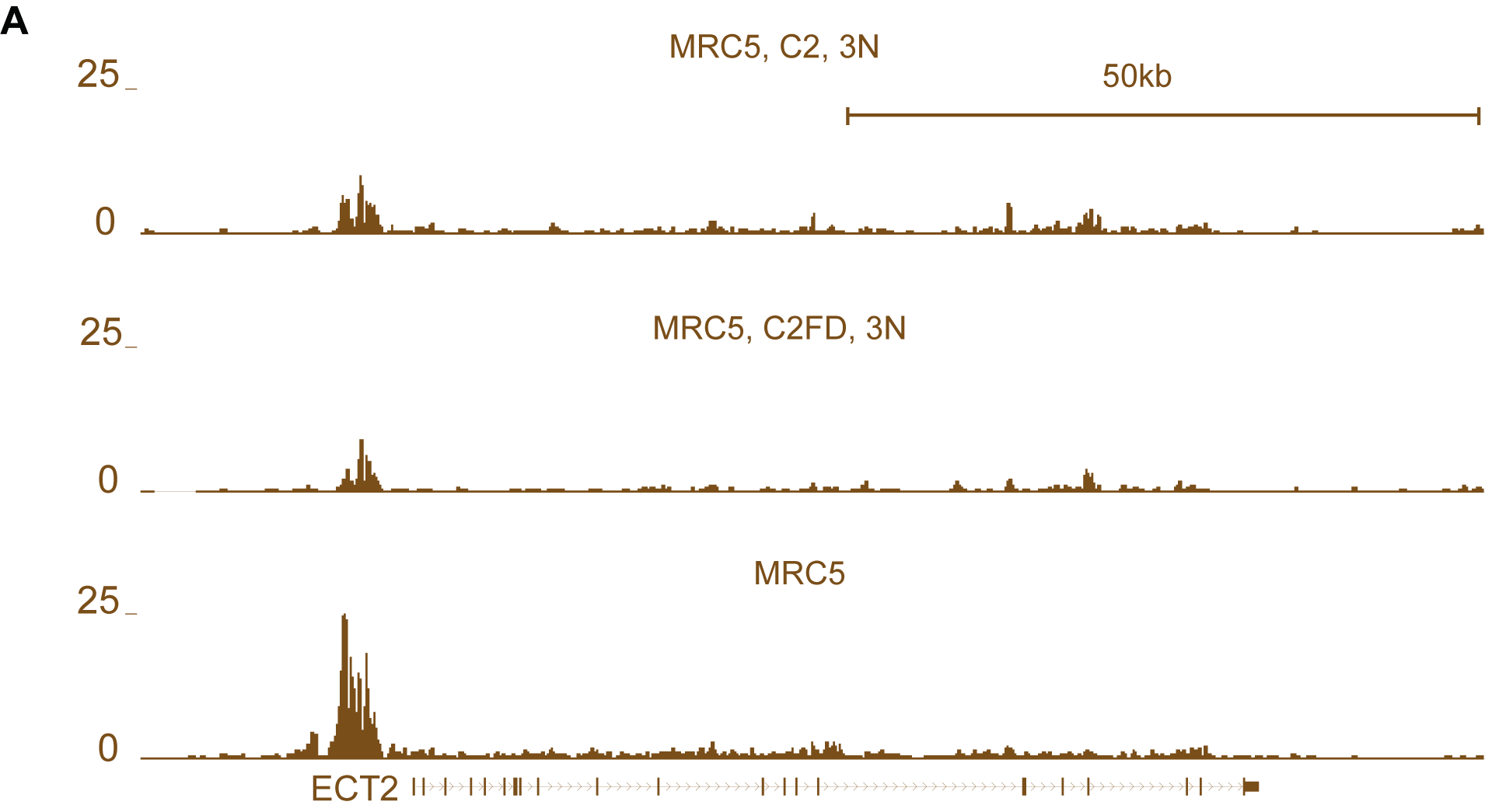

Supplement: Supplementary file 5 — FIGURES5 NGN2 can reduce the acetylation level of the ect2 gene at the promoter region. Histone h3 acetyl k27 chip‐seq in mrc5 cell line at 3 dpi with or without ngn2 infection (3 n, 3 days postinfection of NGN2). [file CNS-30-e70075-s003.tif]

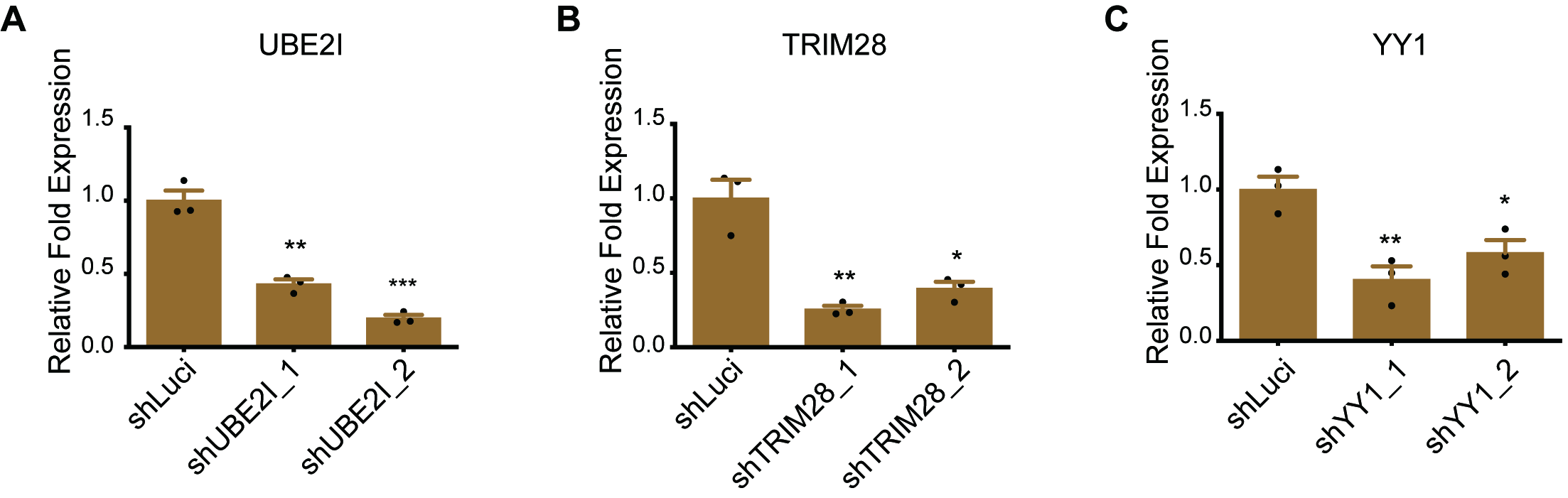

Supplement: Supplementary file 6 — FIGURES6 Knockdown the mRNA expression level of UBE2I, RIM28, and YY1 by shRNA. (A) qRT‐PCR analysis for shUBE2I knockdown efficiency at 3 dpi in U251 cell line (mean ± SEM; n = 3; **p < 0.01 ***p < 0.001). (B) qRT‐PCR analysis for shTRIM28 knockdown efficiency at 3 dpi in U251 cell line (mean ± SEM; n = 3; *p < 0.05 **p < 0.01). (C) qRT‐PCR analysis for shYY1 knockdown efficiency at 3 dpi in U251 cell line (mean ± SEM; n = 3; *p < 0.05 **p < 0.01). [file CNS-30-e70075-s004.tif]

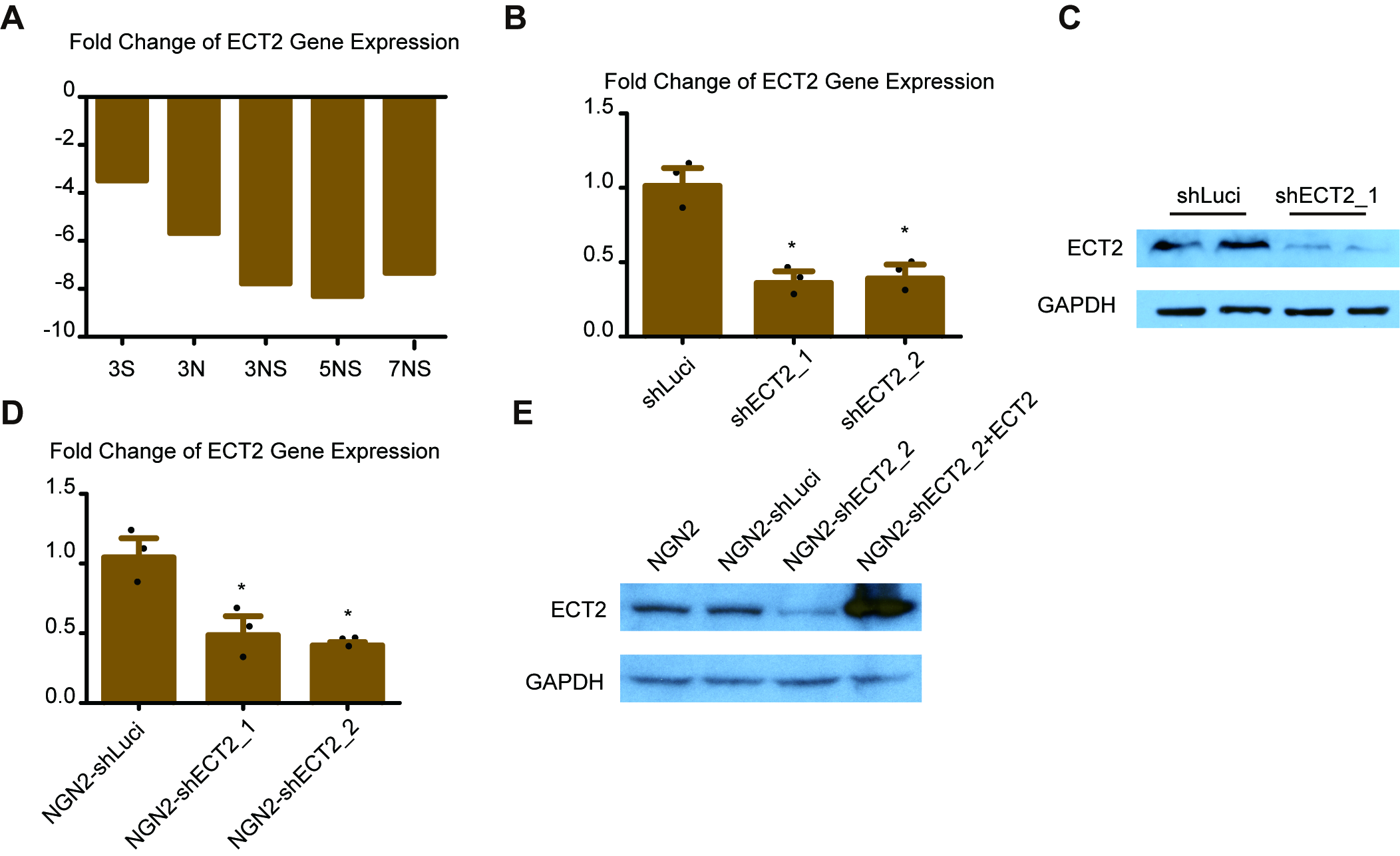

Supplement: Supplementary file 7 — FIGURES7 Confirmation of ECT2 Expression. (A) RNA‐seq data of ECT2 mRNA expression levels in different groups (n = 3). (B) qRT‐PCR analysis for sh_ECT2 knockdown efficiency at 3 dpi in u251 cell line (mean ± SEM; n = 3; *p < 0.05). (C) Western blot analysis for shECT2_1 knockdown efficiency. (D) qRT‐PCR analysis for NGN2‐shECT2 knockdown efficiency at 3 dpi in u251 cell line (mean ± SEM; n = 3; *p < 0.05). (E) Western blot analysis for NGN2‐shECT2_2 knockdown efficiency and ECT2 overexpression. [file CNS-30-e70075-s006.tif]
